# Scientific knowledge is possible with small-sample classification

**DOI:** 10.1186/1687-4153-2013-10

**Published:** 2013-08-20

**Authors:** Edward R Dougherty, Lori A Dalton

**Affiliations:** 1 Department of Electrical and Computer Engineering, Texas A&M University, College Station, TX 77843, USA; 2 Computational Biology Division, Translational Genomics Research Institute, Phoenix, AZ 85004, USA; 3 Department of Electrical and Computer Engineering, The Ohio State University, Columbus, OH 43210, USA

## Abstract

A typical small-sample biomarker classification paper discriminates between types of pathology based on, say, 30,000 genes and a small labeled sample of less than 100 points. Some classification rule is used to design the classifier from this data, but we are given no good reason or conditions under which this algorithm should perform well. An error estimation rule is used to estimate the classification error on the population using the same data, but once again we are given no good reason or conditions under which this error estimator should produce a good estimate, and thus we do not know how well the classifier should be expected to perform. In fact, virtually, in all such papers the error estimate is expected to be highly inaccurate. In short, we are given no justification for any claims.

Given the ubiquity of vacuous small-sample classification papers in the literature, one could easily conclude that scientific knowledge is impossible in small-sample settings. It is not that thousands of papers overtly claim that scientific knowledge is impossible in regard to their content; rather, it is that they utilize methods that preclude scientific knowledge. In this paper, we argue to the contrary that scientific knowledge in small-sample classification is possible provided there is sufficient prior knowledge. A natural way to proceed, discussed herein, is via a paradigm for pattern recognition in which we incorporate prior knowledge in the whole classification procedure (classifier design and error estimation), optimize each step of the procedure given available information, and obtain theoretical measures of performance for both classifiers and error estimators, the latter being the critical epistemological issue. In sum, we can achieve scientific validation for a proposed small-sample classifier and its error estimate.

## Review

### Introduction

It is implicit in the title of this paper that one can entertain the possibility that scientific knowledge is impossible with small-sample classification. In fact, not only might one entertain this impossibility, but perusal of the related literature would most likely lead one to seriously consider that impossibility. It is not that thousands of papers overtly claim that scientific knowledge is impossible with regards to their content; rather, it is that they utilize methods that, *ipso facto*, cannot lead to knowledge. Even though it appears to be almost universally, if tacitly, assumed that scientific knowledge is impossible with small-sample classification - otherwise, why do so many not aspire to such knowledge - we argue to the contrary in this paper that scientific knowledge is possible. But before we make our case, let us examine in more detail why the literature may lead one to believe otherwise.

Consider the following common motif for a small-sample-classification paper, for instance, one proposing a classifier based on gene expression to discriminate types of pathology, stages of a disease, duration of survival, or some other phenotypic difference. Beginning with 30,000 features (genes) and less than 100 labeled sample points (microarrays), some classification rule (algorithm) is selected, perhaps an old one or a new one proposed in the paper. We are given no good reason why this algorithm should perform well. The classification rule is applied to the data and, using the same data, an error estimation rule is used to estimate the classification error on the population, meaning in practice the error rate on future observations. Once again, we are given no good reason why this error estimator should produce a good estimate; in fact, virtually, in all such papers, from what we know about the error estimation rule we would expect the estimate to be inaccurate. At this point, one of two claims is made. If the classification rule is a well-known rule and the purpose of the paper is to produce a classifier for application (say, a biomarker panel), we are told that the authors have achieved their goal of finding such a classifier and its accuracy is validated by the error estimate. If, on the other hand, the purpose is to devise a new classification rule, we are told that the efficacy of the new rule has been validated by its performance, as measured by the error estimate or, by several such error estimates on several different data sets. In either case, we are given no justification for the validation claim. Moreover, in the second case, we are not told the conditions under which the classification rule should be expected to perform well or how well it should be expected to perform.

Amid all of this vacuity, perhaps the reporting of error estimates whose accuracy is a complete mystery is the most puzzling from a scientific perspective. To borrow a metaphor [1], one can imagine Harold Cramér leisurely sailing on the Baltic off the coast of Stockholm, taking in the sights and sounds of the sea, when suddenly a gene-expression classifier to detect prostate cancer pops into his head. No classification rule has been applied, nor is that necessary. All that matters is that Cramér’s imagination has produced a classifier that operates on the feature-label distribution of interest with a sufficiently small error rate. Since scientific validity depends on the predictive capacity of a model, while an appropriate classification rule is certainly beneficial to classifier design, epistemologically, the error rate is paramount. Were we to know the feature-label distribution of interest, we could exactly determine the error rate of the proposed classifier. Absent knowledge of the feature-label distribution, the actual error must be estimated from data and the accuracy of the estimate judged from the performance of the error estimation rule employed. Consequently, any paper that applies an error estimation rule without providing a performance characterization relevant to the data at hand is scientifically vacuous. Given the near universality of vacuous small-sample classification papers in the literature, one could easily reach the conclusion that scientific knowledge is impossible in small-sample settings. Of course, this would beg the question of why people are writing vacuous papers and why journals are publishing them. Since the latter are sociological questions, they are outside the domain of the current paper. We will focus on the scientific issues.

### Epistemological digression

Before proceeding, we digress momentarily for some very brief comments regarding scientific epistemology (referring to [2] for a comprehensive treatise and to [3] for a discussion aimed at biology and including classifier validity). Our aim is narrow, simply to emphasize the role of prediction in scientific knowledge, not to indulge in broad philosophical issues.

A scientific theory consists of two parts: (1) a *mathematical model* composed of symbols (variables and relations between the variables), and (2) a set of *operational definitions* that relate the symbols to data. A mathematical model alone does not constitute a scientific theory. The formal mathematical structure must yield experimental predictions in accord with experimental observations. As put succinctly by Richard Feynman, “It is whether or not the theory gives predictions that agree with experiment. It is not a question of whether a theory is philosophically delightful, or easy to understand, or perfectly reasonable from the point of view of common sense” [4]. Model validity is characterized by predictive relations, without which the model lacks empirical content. Validation requires that the symbols be tied to observations by some semantic rules that relate not necessarily to the general principles of the mathematical model themselves but to conclusions drawn from the principles. There must be a clearly defined tie between the mathematical model and experimental methodology. Philipp Frank writes, “Reichenbach had explicitly pointed out that what is needed is a bridge between the symbolic system of axioms and the protocols of the laboratory. But the nature of this bridge had been only vaguely described. Bridgman was the first who said precisely that these *relations of coordination* consist in the description of physical operations. He called them, therefore, *operational definitions*” [5]. Elsewhere, we have written, “Operational definitions are required, but their exact formulation in a given circumstance is left open. Their specification constitutes an epistemological issue that must be addressed in mathematical (including logical) statements. Absent such a specification, a purported scientific theory is meaningless” [6].

The validity of a scientific theory depends on the choice of validity criteria and the mathematical properties of those criteria. The observational measurements and the manner in which they are to be compared to the mathematical model must be formally specified. The validity of a theory is relative to this specification, but what is not at issue is the necessity of a set of relations tying the model to operational measurements. Formal specification is mandatory and this necessarily takes the form of mathematical (including logical) statements. Formal specification is especially important in stochastic settings where experimental outcomes reflect the randomness of the stochastic system so that one must carefully define how the outcomes are to be interpreted.

Story telling and intuitive arguments cannot suffice. Not only is complex-system behavior often unintuitive, but stochastic processes and statistics often contradict naïve probabilistic notions gathered from simple experiments like rolling dice. Perhaps even worse is an appeal to pretty pictures drawn with computer software. The literature abounds with data partitioned according to some clustering algorithm whose partitioning performance is unknown or, even more strangely, justified by some “validation index” that is poorly, if at all, correlated with the error rate of the clustering algorithm [7]. The pretty pictures are usually multi-colored and augmented with all kinds of attractive-looking symbols. They are inevitably followed by some anecdotal commentary. Although all of this may be delightful, it is scientifically meaningless. Putting the artistic touches and enormous calculations aside, all we are presented with is a radical empiricism. Is there any knowledge here? Hans Reichenbach answers, “A mere report of relations observed in the past cannot be called knowledge. If knowledge is to reveal objective relations of physical objects, it must include reliable predictions. A radical empiricism, therefore, denies the possibility of knowledge” [2]. A collection of measurements together with a commentary on the measurements is not scientific knowledge. Indeed, the entire approach “denies the possibility of knowledge,” so that its adoption constitutes a declaration of meaninglessness.

### Classification error

For two-class classification, the population is characterized by a feature-label distribution *F* for a random pair (**X**,*Y*), where **X** is a vector of *D* features and *Y* is the binary label, 0 or 1, of the class containing **X**. A classifier is a function, *ψ*, which assigns a binary label, *ψ*(**X**), to each feature vector. The error, *ε*[*ψ*], of *ψ* is the probability, *P*(*ψ*(**X**)≠*Y*), that *ψ* yields an erroneous label. A classifier with minimum error among all classifiers is known as a *Bayes classifier* for the feature-label distribution. The minimum error is called the *Bayes error*. Epistemologically, the error is the key issue since it quantifies the predictive capacity of the classifier.

Abstractly, any pair $ℳ=(\psi ,\varepsilon_{\psi })$ composed of a function $\psi :{\mathbb{R}}^{D}\to \{0,1\}$ and a real number *ε*_*ψ*_∈ [0,1] constitutes a *classifier model*, with *ε*_*ψ*_ being simply a number, not necessarily specifying an actual error probability corresponding to *ψ*. ℳ becomes a scientific model when it is applied to a feature-label distribution. In practice, the feature-label distribution is unknown and a *classification ruleΨ*_*n*_ is used to design a classifier *ψ*_*n*_ from a random sample *S*_*n*_={(**X**_1_,*Y*_1_),(**X**_2_,*Y*_2_),…,(**X**_*n*_,*Y*_*n*_)} of pairs drawn from the feature-label distribution. Note that a classification rule is a sequence of rules depending on the sample size *n*. If feature selection is involved, then it is part of the classification rule. A designed classifier produces a classifier model, namely, (*ψ*_*n*_,*ε*[*ψ*_*n*_]). Since the true classifier error *ε*[*ψ*_*n*_] depends on the feature-label distribution, which is unknown, *ε*[*ψ*_*n*_] is unknown. The true error must be estimated by an *estimation rule*, *Ξ*_*n*_. Thus, the random sample *S*_*n*_ yields a classifier *ψ*_*n*_=*Ψ*_*n*_(*S*_*n*_) and an error estimate $\hat{\varepsilon }[\psi_{n}]=\Xi_{n}(S_{n})$, which together constitute a classifier model $\left(\psi_{n},\hat{\varepsilon }[\psi_{n}]\right)$. Overall, classifier design involves a *rule model* (*Ψ*_*n*_,*Ξ*_*n*_) used to determine a sample-dependent classifier model $\left(\psi_{n},\hat{\varepsilon }[\psi_{n}]\right)$. Both (*ψ*_*n*_,*ε*[*ψ*_*n*_]) and $\left(\psi_{n},\hat{\varepsilon }[\psi_{n}]\right)$ are random pairs relative to the sampling distribution.

Given a feature-label distribution, error estimation accuracy is commonly measured by the *mean-square error* (*MSE*), defined by $\mathrm{MSE}(\hat{\varepsilon })=E[{\left(\hat{\varepsilon }-\varepsilon \right)}^{2}]$, where for notational ease we denote *ε*[*ψ*_*n*_] and $\hat{\varepsilon }[\psi_{n}]$ by *ε* and $\hat{\varepsilon }$, respectively, or, equivalently, by the square root of the MSE, known as the *root-mean-square* (*RMS*). The expectation used here is relative to the sampling distribution induced by the feature-label distribution. The MSE is decomposed into the bias, $\text{Bias}(\hat{\varepsilon })=E[\hat{\varepsilon }-\varepsilon ]$, of the error estimator relative to the true error, and the deviation variance, ${\text{Var}}_{\text{dev}}(\hat{\varepsilon })=\text{Var}(\hat{\varepsilon }-\varepsilon )$, by

(1) $$\text{MSE}(\hat{\varepsilon })={\text{Var}}_{\text{dev}}(\hat{\varepsilon })+\text{Bias}{\left(\hat{\varepsilon }\right)}^{2}.$$

When a large amount of data is available, the sample can be split into independent training and test sets, the classifier being designed on the training data and its error being estimated by the proportion of errors on the test data, which is known as the holdout estimator. For holdout, we have the distribution-free bound $\text{RMS}({\hat{\varepsilon }}_{\mathrm{holdout}}|S_{n-m},F)\leq 1/\sqrt{4m}$, where *m* is the size of the test sample, *S*_*n*−*m*_ is the training sample and *F* is any feature-label distribution [8]. $\text{RMS}(\hat{\varepsilon }|Z)$ indicates that the expectation in the RMS is conditioned on the random vector *Z*. But when data are limited, the sample cannot be split without leaving too little data to design a good classifier. Hence, training and error estimation must take place on the same data set.

The consequences of training-set error estimation are readily explained by the following formula for the deviation variance:

(2) $${\text{Var}}_{\text{dev}}(\hat{\varepsilon })=\sigma_{\hat{\varepsilon }}^{2}+\sigma_{\varepsilon }^{2}-2\rho \sigma_{\hat{\varepsilon }}\sigma_{\varepsilon },$$

where $\sigma_{\hat{\varepsilon }}^{2},\sigma_{\varepsilon }^{2},$ and *ρ* are the variance of the error estimate, the variance of the error, and the correlation between the estimated and true errors, respectively. The deviation variance is driven down by small variances or a correlation coefficient near 1.

Consider the popular cross-validation error estimator. For it, the error is estimated on the training data by randomly splitting the training data into *k* folds (subsets), $S_{n}^{i}$, for *i*=1,2,...,*k*, training *k* classifiers on $S_{n}-S_{n}^{i}$, for *i*=1,2,...,*k*, calculating the proportion of errors of each designed classifier on the appropriate left-out fold, and then averaging these proportions to obtain the cross-validation estimate of the originally designed classifier. Various enhancements are made, such as by repeating the process some number of times and averaging. Letting *k* = *n* yields the leave-one-out estimator. The problem with cross-validation is evident from (2): for small samples, it has large variance and little correlation with the true error. Hence, although with small folds, cross-validation does not suffer too much from bias, it typically has large deviation variance.

**Figure 1:**
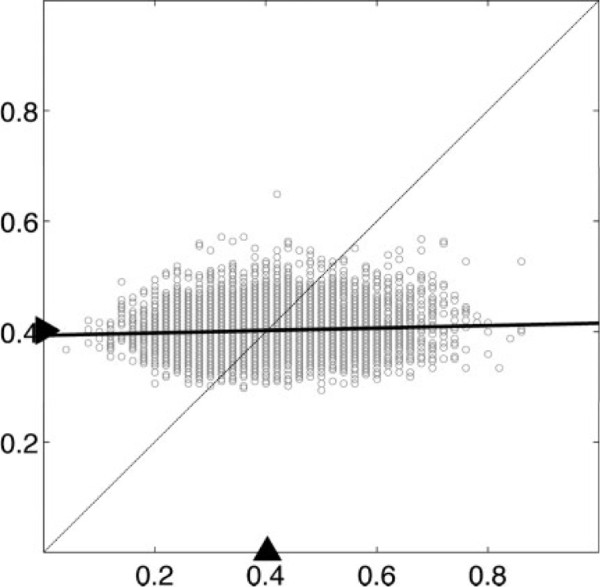
**Linear regression between cross-validation and the true error.** Scatter plot and linear regression for cross-validation (horizontal axis) and the true error (vertical axis) with sample size 50 for linear discrimination between two classes of breast cancer patients.

To illustrate the matter, we reproduce an example from [9] based on real patient data from a study involving microarrays prepared with RNA from breast tumor specimens from 295 patients, 115 and 180 belonging to the good-prognosis and poor-prognosis classes, respectively. The dataset is reduced to the 2,000 genes with highest variance, these are reduced to 10 via *t* test feature selection, and a classifier is designed using linear discriminant analysis (LDA). In the simulations, the data are split into two sets. The first set, consisting of 50 examples drawn without replacement from the full dataset, is used for both training and error estimation via leave-one-out cross-validation. The remaining examples are used as a hold-out test set to get an accurate estimate of the true error, which is taken as the true error. There is an assumption that such a hold-out size will give an accurate estimate of the true error. This procedure is repeated 10,000 times. Figure 1 shows the scatter plot for the pairs of true and estimated errors, along with the linear regression of the true error on the estimated error. The means are shown on the axes. What we observe is typical for small samples: large variance and negligible regression between the true and estimated errors [10]. Indeed, one even sees negatively sloping regression lines for cross-validation and bootstrap (another resampling error estimator), and negative correlation between the true and cross-validation estimated errors has been mathematically demonstrated in some basic models [11]. Such error estimates are worthless and can lead to a huge waste of resources in trying to reproduce them [9].

### RMS bounds

Suppose a sample is collected, a classification rule *Ψ*_*n*_ applied, and the classifier error estimated by an error-estimation rule *Ξ*_*n*_ to arrive at the classifier model $\left(\psi_{n},\hat{\varepsilon }[\psi_{n}]\right)$. If no assumptions are posited regarding the feature-label distribution, then the entire procedure is completely distribution-free. There are three possibilities. First, if no validity criterion is specified, then the classifier model is *ipso facto* epistemologically meaningless. Second, if a validity criterion is specified, say RMS, and no distribution-free results are known about the RMS for *Ψ*_*n*_ and *Ξ*_*n*_, then again the model is meaningless. Third, if there exist distribution-free RMS bounds concerning *Ψ*_*n*_ and *Ξ*_*n*_, then these bounds can, in principle, be used to quantify the performance of the error estimator and thereby quantify model validity.

Regarding the third possibility, the following is an example of a distribution-free RMS bound for the leave-one-out error estimator with the discrete histogram rule and tie-breaking in the direction of class 0 [8]:

(3) $$\text{RMS}({\hat{\varepsilon }}_{\text{loo}}|F)\leq \sqrt{\frac{1+6/e}{n}+\frac{6}{\sqrt{\pi \left(n-1\right)}}},$$

where *F* is any feature-label distribution. Although this bound holds for all distributions, it is useless for small samples: for *n*=200 this bound is 0.506. In general, there are very few cases in which distribution-free bounds are known and, when they are known, they are useless for small samples.

Distribution-based bounds are needed. These require knowledge of the RMS, which means knowledge concerning the second-order moments of the joint distribution between the true and estimated errors. More generally, to fully understand an error estimator we need to know its joint distribution with the true error. Oddly, this problem has historically been ignored in pattern recognition, notwithstanding the fact that error estimation is the epistemological ground for classification. Going back to the 1970s there were some results on the mean and variance of some error estimators for the Gaussian model using LDA. In 1966, Hills obtained the expected value of the resubstitution and plug-in estimators in the univariate model with known common variance [12]. The resubstitution estimate is simply a count of the classification errors on the training data and the plug-in estimate is found by using the data to estimate the feature-label distribution and then finding the error of the designed classifier on the estimated distribution. In 1972, Foley obtained the expected value of resubstitution in the multivariate model with known common covariance matrix [13]. In 1973, Sorum derived results for the expected value and variance for both resubstitution and leave-one-out in the univariate model with known common variance [14]. In 1973, McLachlan derived an asymptotic representation for the expected value of resubstitution in the multivariate model with unknown common covariance matrix [15]. In 1975, Moran obtained new results for the expected value of resubstitution and plug-in for the multivariate model with known covariance matrix [16]. In 1977, Goldstein and Wolf obtained the expected value of resubstitution for multinomial discrimination [17]. Following the latter, there was a gap of 15 years before Davison and Hall derived asymptotic representations for the expected value and variance of bootstrap and leave-one-out in the univariate Gaussian model with unknown and possibly different covariances [18]. This is the only paper we know of providing analytic results for moments of common error estimators between 1977 and 2005. None of these papers provided representation of the joint distribution or representation of second-order mixed moments, which are needed for the RMS.

This problem has only recently been addressed beginning in 2005, in particular, for the resubstitution and leave-one-out estimators. For the multinomial model, complete enumeration was used to obtain the marginal distributions for the error estimators [11] and then the joint distributions [19]. Exact closed-form representations for second-order moments, including the mixed moments, were obtained, thereby obtaining exact RMS representations for both estimators [11]. For the Gaussian model using LDA in 2009, we obtained the exact marginal distributions for both estimators in the univariate model (known but not necessarily equal class variances) and approximations in the multivariate model (known and equal class covariance matrices) [20]. Subsequently, these were extended to the joint distributions for the true and estimated errors in a Gaussian model [21]. Recently exact closed-form representations for the second-order moments in the univariate model without assuming equal covariances were discovered, thereby providing exact expression of the RMS for both estimators [22]. Moreover, double asymptotic representations for the second-order moments in the multivariate model, sample size and dimension approaching infinity at a fixed rate between the two, were found, thereby providing double asymptotic expressions for the RMS [23]. Finite sample approximations from the double asymptotic method have been shown to possess better accuracy than various simple asymptotic representations (although much more work is needed on this issue) [24,25].

### Validity

Let us now consider validity. An obvious way to proceed would be to say that a classifier model (*ψ*,*ε*_*ψ*_) is valid for the feature-label distribution *F* to the extent that *ε*_*ψ*_ approximates the classifier error, *ε*[*ψ*], on *F*, where the degree of approximation is measured by some distance between *ε*_*ψ*_ and *ε*[*ψ*]. For a classifier *ψ*_*n*_ designed from a specific sample, this would mean that we want to measure some distance between *ε*=*ε*[*ψ*_*n*_] and $\hat{\varepsilon }=\hat{\varepsilon }[\psi_{n}]$, say $\left|\varepsilon -\hat{\varepsilon }\right|$. To do this, we would have to know the true error and to know that we would need to know *F*. But if we knew *F*, we would use the Bayes classifier and would not need to design a classifier from sample data. Since it is the precision of the error estimate that is of consequence, a natural way to proceed would be to characterize validity in terms of the precision of the error estimator $\hat{\varepsilon }[\psi_{n}]=\Xi_{n}(S_{n})$ as an estimator of *ε*[*ψ*_*n*_], say by $\text{RMS}(\hat{\varepsilon })$. This makes sense because both the true and estimated errors are random functions of the sample and the RMS measures their closeness across the sampling distribution. But again there is a catch: the RMS depends on *F*, which we do not know. Thus, given the sample without knowledge of *F*, we cannot compute the RMS.

**Figure 2:**
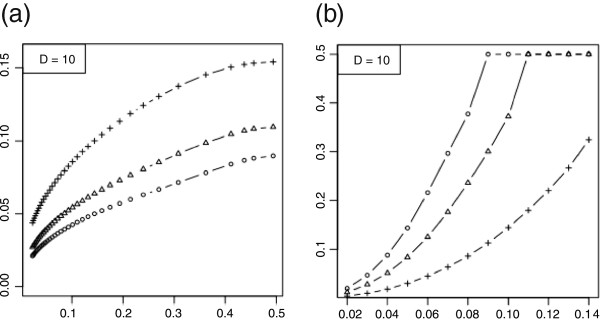
**RMS and*maxBayes(λ)*.(a)** RMS (*y*-axis) as a function of the Bayes error (*x*-axis) for leave-one-out with dimension *D*=10 and sample sizes *n*=20 (plus sign), 40 (triangle), 60 (circle); **(b)** maxBayes(*λ*) curves corresponding to the RMS curves in part **(a)**.

To proceed, prior knowledge is required, in the sense that we need to assume that the actual (unknown) feature-label distribution belongs to some *uncertainty class*, U, of feature-label distributions. Once RMS representations have been obtained for feature-label distributions in U, distribution-based RMS bounds follow: $\text{RMS}(\hat{\varepsilon })\leq \underset{G\in U}{\max}\text{RMS}(\hat{\varepsilon }|G)$, where $\text{RMS}(\hat{\varepsilon }|G)$ is the RMS of the error estimator under the assumption that the feature-label distribution is *G*. We do not know the actual feature-label distribution precisely, but prior knowledge allows us to bound the RMS. For instance, consider using LDA with a feature-label distribution having two equally probable Gaussian class-conditional densities sharing a known covariance matrix. For this model the Bayes error is a one-to-one decreasing function of the distance, *m*, between the means. Figure 2a shows the RMS to be a one-to-one increasing function of the Bayes error for leave-one-out for dimension *D*=10 and sample sizes *n*=20,40,60, the RMS and Bayes errors being on the *y* and *x* axes, respectively.

Assuming a parameterized model in which the RMS is an increasing function of the Bayes error, *ε*_bay_, we can pose the following question: Given sample size *n* and *λ*>0, what is the maximum value, maxBayes(*λ*), of the Bayes error such that $\text{RMS}(\hat{\varepsilon })\leq \lambda$? If RMS is the measure of validity and *λ* represents the largest acceptable RMS for the classifier model to be considered meaningful, then the epistemological requirement is characterized by maxBayes(*λ*). Given the relationship between model parameters and the Bayes error, the inequality *ε*_bay_≤maxBayes(*λ*) can be solved in terms of the parameters to arrive at a necessary modeling assumption. In the preceding Gaussian example, since *ε*_bay_ is a decreasing function of *m*, we obtain an inequality *m*≥*m*(*λ*). Figure 2b shows the maxBayes(*λ*) curves corresponding to the RMS curves in Figure 2a [26]. These curves show that, assuming Gaussian class-conditional densities and a known common covariance matrix, further assumptions must be made to insure that the RMS is sufficiently small to make the classifier model meaningful.

To have scientific content, small-sample classification requires prior knowledge. Regarding the feature-label distribution, there are two extremes: (1) the feature-label distribution is known, in which case the entire classification problem collapses to finding the Bayes classifier and Bayes error, so there is no classifier design or error estimation issue; and (2) the uncertainty class consists of all feature-label distributions, the distribution-free case, and we typically have no bound, or one that is too loose for practice. In the middle ground, there is a trade-off between the size of the uncertainty class and the size of the sample. The uncertainty class must be sufficiently constrained (equivalently, the prior knowledge must be sufficiently great) that an acceptable bound can be achieved with an acceptable sample size.

### MMSE error estimation

Given that one needs a distributional model to achieve useful performance bounds for classifier error estimation, an obvious course of action is to find or define a prior over the uncertainty class of feature-label distributions, and then find an optimal minimum-mean-square-error (MMSE) error estimator relative to that class [27]. This results in a Bayesian approach with the uncertainty class being given a prior distribution and the data being used to construct a posterior distribution, which quantifies everything we know about the feature-label distribution. Benefits of the Bayesian approach are (1) we can incorporate prior knowledge in the whole classification procedure (classifier design and error estimation), which, as we have argued above, is desperately needed in a small-sample setting where the data provide only a meager amount of information; (2) given the mathematical framework, we can optimize each step of the procedure, further addressing the poor performance suffered in small samples; and (3) we can obtain theoretical measures of the performance for both arbitrary classifiers (via the MMSE error estimator) and arbitrary error estimators (via the sample conditioned MSE), perhaps the most important advantage epistemologically. We begin with an overview of optimal MMSE error estimation.

Assume that a sample point has a prior probability *c* of coming from class 0, and that the class-0 conditional distribution is parameterized by *θ*_0_ and class 1 is parameterized by *θ*_1_. Considering both classes, our model is completely parameterized by *θ*={*c*,*θ*_0_,*θ*_1_}. Given a random sample, *S*_*n*_, we design a classifier *ψ*_*n*_ and wish to minimize the MSE between its true error, *ε* (a function of *θ* and *ψ*_*n*_), and an error estimate, $\hat{\varepsilon }$ (a function of *S*_*n*_ and *ψ*_*n*_). A key realization is that the expectation in the MSE may now be taken over the uncertainty class conditioned on the observed sample, rather than over the sampling distribution for a fixed (unknown) feature-label distribution. The MMSE error estimator is thus the expected true error, $\hat{\varepsilon }(\psi_{n},S_{n})=E_{\theta }[\varepsilon (\psi_{n},\theta )|S_{n}]$. The expectation given the sample is over the posterior density of *θ*, denoted by *π*^∗^(*θ*). Thus, we write the Bayesian MMSE error estimator with the shorthand $\hat{\varepsilon }=E_{\pi^{*}}[\varepsilon ]$.

The Bayesian error estimate is not guaranteed to be the optimal error estimate for any particular feature-label distribution but optimal for a given sample, and assuming the parameterized model and prior probabilities, it is both optimal on average with respect to MSE and unbiased when averaged over all parameters and samples. These implications apply for any classification rule as long as the classifier is fixed given the sample. To facilitate analytic representations, we assume *c*, *θ*_0_ and *θ*_1_ are all mutually independent prior to observing the data. Denote the marginal priors of *c*, *θ*_0_ and *θ*_1_ by *π*(*c*), *π*(*θ*_0_) and *π*(*θ*_1_), respectively, and suppose data are used to find each posterior, *π*^∗^(*c*), *π*^∗^(*θ*_0_) and *π*^∗^(*θ*_1_), respectively. Independence is preserved, i.e., *π*^∗^(*c*,*θ*_0_,*θ*_1_)=*π*^∗^(*c*)*π*^∗^(*θ*_0_)*π*^∗^(*θ*_1_) [27].

If *ψ*_*n*_ is a trained classifier given by *ψ*_*n*_(**x**)=0 if **x**∈*R*_0_ and *ψ*_*n*_(**x**)=1 if **x**∈*R*_1_, where *R*_0_ and *R*_1_ are measurable sets partitioning the sample space, then the true error of *ψ*_*n*_ under the distribution parameterized by *θ* may be decomposed as

(4) $$\begin{array}{lll} \varepsilon (\psi_{n},\theta ) & =c\int_{R_{1}}f_{\theta_{0}}\left(x|0\right)dx+(1-c)\underset{R_{0}}{\int }f_{\theta_{1}}\left(x|1\right)dx\;\\ & =c\varepsilon^{0}(\psi_{n},\theta_{0})+(1-c)\varepsilon^{1}(\psi_{n},\theta_{1}),\; & \; \end{array}$$

where $f_{\theta_{y}}\left(x|y\right)$ is the class-*y* conditional density assuming parameter *θ*_*y*_ is true and *ε*^*y*^ is the error contributed by class *y*. Owing to the posterior independence between *c* and *θ*_0_ and between *c* and *θ*_1_, the Bayesian MMSE error estimator can be expressed as [28]

(5) $$\hat{\varepsilon }\left(\psi_{n},S_{n}\right)=E_{\pi^{*}}[c]E_{\pi^{*}}[\varepsilon^{0}]+(1-E_{\pi^{*}}[c])E_{\pi^{*}}[\varepsilon^{1}].$$

With a fixed sample and classifier, and given *θ*_*y*_, the true error, *ε*^*y*^(*ψ*_*n*_,*θ*_*y*_), is deterministic. Thus, letting **Θ**_*y*_ be the parameter space of *θ*_*y*_,

(6) $$E_{\pi^{*}}[\varepsilon^{y}]=\int_{\Theta_{y}}\varepsilon^{y}(\psi_{n},\theta_{y})\pi^{*}(\theta_{y})d\theta_{y}.$$

Just as the true error for a fixed feature-label distribution is found from the class-conditional densities, $f_{\theta_{y}}\left(x|y\right)$, the Bayesian MMSE error estimator for an uncertainty class can be found from *effective class-conditional densities*, which are derived by taking the expectations of the individual class-conditional densities with respect to the posterior distribution,

(7) $$f\left(x|y\right)=\int_{\Theta_{y}}f_{\theta_{y}}\left(x|y\right)\pi^{*}\left(\theta_{y}\right)d\theta_{y}.$$

Specifically, we obtain an equation for the expected true error that parallels that of the true error in (4) [29]:

(8) $$\hat{\varepsilon }\left(\psi_{n},S_{n}\right)\;=\;E_{\pi^{*}}[\;c]\;\int_{R_{1}}\;f\left(x|0\right)dx+(1-E_{\pi^{*}}[\;c])\;\;\int_{R_{0}}\;f\;\left(x|1\right)dx.$$

Application of Bayesian error estimation to real data, in particular gene-expression microarray data, has been addressed in [30]. This work provides C code implementing the Bayesian error estimator for Gaussian distributions and normal-inverse-Wishart priors for both linear classifiers, with exact closed-form representations, and non-linear classifiers, where closed form-solutions are not available and we instead implement a Monte-Carlo approximation. The code and a toolbox of related utilities are publicly available. In [30] we discuss the suitability of a Gaussian model with normal-inverse-Wishart priors for microarray data and propose a feature selection scheme employing a Shapiro-Wilk Gaussianity test to validate Gaussian modeling assumptions. Furthermore, we propose a methodology for calibrating normal-inverse-Wishart priors for microarray data based on a method-of-moments approach using features discarded by the feature-selection scheme.

### Sample-conditioned MSE

The RMS of an error estimator is used to characterize the validity of a classifier model. As we have discussed, if we are in possession of RMS expressions for the feature-label distributions in an uncertainty class, we can bound the RMS, so as to insure a given level of performance. In the case of MMSE error estimation, the priors provide a mathematical framework that can be used for both the analysis of any error estimator and the design of estimators with desirable properties or optimal performance. The posteriors of the distribution parameters imply a (sample-conditioned) distribution on the true classifier error. This randomness in the true error comes from our uncertainty in the underlying feature-label distribution (given the sample). Within the assumed model, this sample-conditioned distribution of the true error contains the full information about error estimator accuracy and we may speak of moments of the true error (for a fixed sample and classifier), in particular the expectation, variance, and sample-conditioned MSE, as opposed to simply the MSE relative to the sampling distribution as in classical error estimation.

Finding the sample-conditioned MSE of MMSE Bayesian error estimators amounts to evaluating the variance of the true error conditioned on the observed sample [28]. The sample-conditioned MSE converges to zero almost surely in both discrete and Gaussian models provided in [31], where closed form expressions for the MSE are available. Further, the exact MSE for arbitrary error estimators falls out naturally in the Bayesian model. That is, if ${\hat{\varepsilon }}_{∙}$ is a constant representing an arbitrary error estimate computed from the sample, then the MSE of ${\hat{\varepsilon }}_{∙}$ can be evaluated directly from that of the Bayesian error estimator:

$$\text{MSE}({\hat{\varepsilon }}_{∙}|S_{n})=\text{MSE}(\hat{\varepsilon }|S_{n})+{\left(\hat{\varepsilon }-{\hat{\varepsilon }}_{∙}\right)}^{2}.$$

$\text{MSE}({\hat{\varepsilon }}_{∙}|S_{n})$, as well as its square root $\text{RMS}({\hat{\varepsilon }}_{∙}|S_{n})$, are minimized when $\hat{\varepsilon }={\hat{\varepsilon }}_{∙}$.

**Figure 3:**
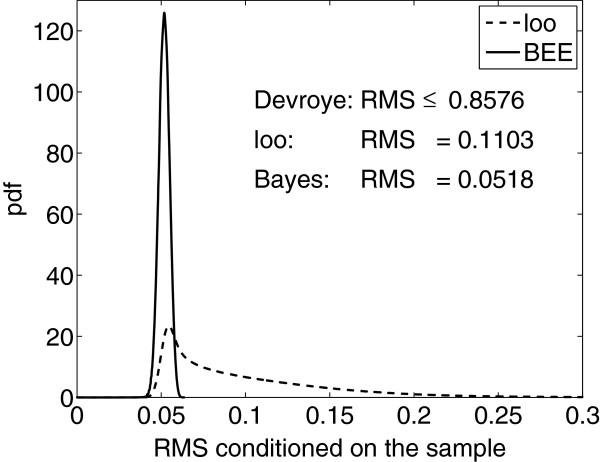
**Sample-conditioned RMS probability densities.** Probability densities for the sample-conditioned RMS of leave-one-out (dashed line) and the Bayesian error estimator (solid line) in a discrete model with *b*=16 bins, prior probability *c*=0.5, *n*=30 training points, and an average true error of 0.25.

In a classical approach, nothing is known given a sample, whereas in a Bayesian approach, the sample conditions uncertainty in the RMS and different samples may condition it to different extents. Figure 3 shows probability densities of the sample-conditioned RMS for both the leave-one-out estimator and Bayesian error estimator in a discrete model with *b*=16 bins. The simulation generates 10,000 distributions drawn from a prior given in [31] and 1,000 samples from each distribution. The unconditional RMS (averaged over both distributions and samples) for both error estimators is also shown, as well as the distribution-free RMS bound on leave-one-out given in (3). In Figure 3, the RMS of the Bayesian error estimator tends to be very close to 0.05 whereas the leave-one-out error estimator has a long tail with substantial mass between 0.05 and 0.2, demonstrating that different samples can condition the RMS to a very significant extent. In addition, the unconditional RMS of the Bayesian error estimator is less than half that of leave-one-out, while Devroye’s distribution-free bound on the unconditional RMS is too loose to be useful. Hence, not only does a Bayesian framework permit us to obtain an optimal error estimator and its RMS conditioned on the sample, but performance improvement can be significant.

In [31], a bound on the sample-conditioned RMS of the Bayesian error estimator is provided for the discrete model. With any classifier, beta priors on *c* and Dirichlet priors on the bin probabilities satisfying mild conditions, and given a sample *S*_*n*_, $\text{RMS}({\hat{\varepsilon }}_{\text{BEE}}|S_{n})\leq 1/\sqrt{4n}$. For comparison, consider the holdout bound $\text{RMS}({\hat{\varepsilon }}_{\mathrm{holdout}}|S_{n-m},F)\leq 1/\sqrt{4m}$, where *m* is the size of the test sample. Both bounds still hold if we remove the conditioning, and in this way they become comparable. Since $1/\sqrt{4n}\leq 1/\sqrt{4m}$, under a Bayesian model not only does using the full sample to train the classifier result in a lower true error, but we expect to achieve better RMS performance using training-data error estimation than we would by holding out the entire sample for error estimation. This is a testament to the power of modeling.

### Optimal classification

Since prior knowledge is required to obtain a good error estimate in small-sample settings, an obvious course of action would be to utilize that knowledge for classifier design [29,32]. Whereas ordinary *Bayes classifiers* minimize the misclassification probability when the underlying distributions are known, *optimal Bayesian classification* trains a classifier from data assuming the feature-label distribution is contained in a family parameterized by *θ*∈**Θ** with some assumed prior density over the states. Formally, we define an optimal Bayesian classifier, *ψ*_OBC_, as any classifier satisfying

(9) $$E_{\pi^{*}}\left[\varepsilon (\psi_{\text{OBC}},\theta )\right]\leq E_{\pi^{*}}\left[\varepsilon (\psi ,\theta )\right]$$

for all ψ∈C, where C is an arbitrary family of classifiers. Under the Bayesian framework, this is equivalent to minimizing the probability of error as follows:

(10) $$\begin{array}{l} P\left(\psi_{n}\left(X\right)\neq Y|S_{n}\right)=E_{\pi^{*}}\left[P\left(\psi_{n}\left(X\right)\neq Y|\theta ,S_{n}\right)\right]\;\\ \;=E_{\pi^{*}}\left[\varepsilon (\psi_{n},\theta )\right]\;\\ \;=\hat{\varepsilon }\left(\psi_{n},S_{n}\right).\; \end{array}$$

An optimal Bayesian classifier can be found by brute force using the closed form solutions for the expected true error (the Bayesian error estimator), when available. However, if C is the set of all classifiers (with measurable decision regions), then an optimal Bayesian classifier can be found analogously to Bayes classification for a fixed distribution using the effective class-conditional densities. To wit, we can realize an optimal solution without explicitly finding the error for every classifier because the solution can be found pointwise. Specifically, an optimal Bayesian classifier, *ψ*_OBC_, satisfying (9) for all ψ∈C, the set of all classifiers with measurable decision regions, exists and is given pointwise by [29]

(11) $$\psi_{\text{OBC}}\left(x\right)=\left\{\begin{array}{ll} 0 & \text{if}\;E_{\pi^{*}}[c]f\left(x|0\right)\geq (1-E_{\pi^{*}}[c])f\left(x|1\right),\\ 1 & \text{otherwise}. \end{array}\right)$$

If $E_{\pi^{*}}[c]=0$, then this optimal Bayesian classifier is a constant and always assigns class 1, and if $E_{\pi^{*}}[c]\;=1$ it always assigns class 0. Hence, we will typically assume that $0<E_{\pi^{*}}[c]<1$.

Essentially, the optimal thing to do is to find the Bayes classifier using *f*(**x**|*y*) as the true class-conditional distributions. This is like a plug-in rule, only *f*(**x**|*y*) is not necessarily in the family of distributions $\left\{\;f_{\theta_{y}}(x|y)\right\}$, but some other kind of density that happens to result in the optimal classifier. We find the optimal Bayesian classifier without explicitly evaluating the expected true error, $E_{\pi^{*}}\left[\varepsilon (\psi ,\theta )\right]$, for every possible classifier *ψ*. With regards to both optimal Bayesian classification and Bayesian MMSE error estimation, *f*(**x**|*y*) contains all of the necessary information in the model about the class-conditional distributions and we do not have to deal with the uncertainty class or priors directly. Upon defining a model, we find *f*(**x**|*y*) (which depends on the sample because it depends on *π*^∗^) and then the whole problem is solved by treating *f*(**x**|*y*) as the true distribution: optimal classification, the error estimate of the optimal classifier, and the optimal error estimate for arbitrary classifiers. That being said, there is no short-cut to finding the sample-conditioned MSE via the effective density; indeed, there is no notion of variance in the true error of a fixed classifier under the effective class-conditional densities. Moreover, the approach of using the effective class-conditional densities finds an optimal Bayesian classifier over all possible classifiers. On the other hand, there may be advantages to restricting the space of classifiers, for example, in a Gaussian model one may prefer linear classifiers where closed-form Bayesian error estimators have been found [33].

We will present a Bayesian MMSE classifier for the discrete model, which has already been solved. More generally, what we are proposing is not just a few new classifiers, but a new paradigm in classifier design focused on optimization over a concrete mathematical framework. Furthermore, this work ties Bayesian modeling and the Bayesian error estimator together with the old problem of optimal robust filtering; indeed, in the absence of observations, the optimal Bayesian classifier reduces to the Bayesian robust optimal classifier [32,34].

### Optimal discrete classification

To illustrate concepts in optimal Bayesian classification, we consider discrete classification, in which the sample space is discrete with *b* bins. We let *p*_*i*_ and *q*_*i*_ be the class-conditional probabilities in bin *i* ∈ {1,…,*b*} for class 0 and 1, respectively, and we define *U*_*j*_ and *V*_*j*_ to be the number of sample points observed in bin *j*∈{1,…,*b*} from class 0 and 1, respectively. The class sizes are given by $n_{0}=\sum_{i=1}^{b}U_{i}$ and $n_{1}=\sum_{i=1}^{b}V_{i}$. A general discrete classifier assigns each bin to a class, so *ψ*_*n*_:{1,…,*b*}→{0,1}.

The discrete Bayesian model defines *θ*_0_= [*p*_1_,…,*p*_*b*−1_] and *θ*_1_= [*q*_1_,…,*q*_*b*−1_]. The last bin probabilities are not needed since $p_{b}=1-\sum_{i=1}^{b-1}p_{i}$ and $q_{b}=1-\sum_{i=1}^{b-1}q_{i}$. The parameter space of *θ*_0_ is defined to be the set of a valid bin probabilities, e.g., [*p*_1_,…,*p*_*b*−1_]∈**Θ**_0_ if and only if 0≤*p*_*i*_≤1 for *i*∈{1,…,*b*−1} and $\sum_{i=1}^{b-1}p_{i}\leq 1$. The parameter space **Θ**_1_ is defined similarly. With the parametric model established, we define conjugate Dirichlet priors

(12) $$\pi (\theta_{0})\propto \overset{b}{\underset{i=1}{\prod }}p_{i}^{\alpha_{i}^{0}-1}\;\text{and}\;\pi (\theta_{1})\propto \overset{b}{\underset{i=1}{\prod }}q_{i}^{\alpha_{i}^{1}-1}.$$

For proper priors, the hyperparameters, $\alpha_{i}^{y}$ for *i*∈{1,…,*b*} and *y*∈{0,1}, must be positive, and for uniform priors $\alpha_{i}^{y}=1$ for all *i* and *y*. In this setting, the posteriors are again Dirichlet, and when normalized they are given by

(13) $$\begin{array}{ll} \pi^{*}(\theta_{0}) & =\frac{\Gamma \;\left(n_{0}+\overset{b}{\underset{i=1}{\sum }}\alpha_{i}^{0}\right)}{\overset{b}{\underset{k=1}{\prod }}\Gamma \left(U_{k}+\alpha_{k}^{0}\right)}\overset{b}{\underset{i=1}{\prod }}p_{i}^{U_{i}+\alpha_{i}^{0}-1},\; \end{array}$$

(14) $$\begin{array}{ll} \pi^{*}(\theta_{1}) & =\frac{\Gamma \;\left(n_{1}+\overset{b}{\underset{i=1}{\sum }}\alpha_{i}^{1}\right)}{\overset{b}{\underset{k=1}{\prod }}\Gamma \left(V_{k}+\alpha_{k}^{1}\right)}\overset{b}{\underset{i=1}{\prod }}q_{i}^{V_{i}+\alpha_{i}^{1}-1},\; \end{array}$$

where *Γ* is the Gamma function.

In the discrete model, for *j*∈{1,…,*b*} the effective class-conditional densities can be shown to be equal to

(15) $$f\left(j|0\right)=\frac{U_{j}+\alpha_{j}^{0}}{n_{0}+\overset{b}{\underset{i=1}{\sum }}\alpha_{i}^{0}}\;\text{and}\;\;f\left(j|1\right)=\frac{V_{j}+\alpha_{j}^{1}}{n_{1}+\overset{b}{\underset{i=1}{\sum }}\alpha_{i}^{1}}.$$

*f*(*j*|0) and *f*(*j*|1) may be viewed as effective bin probabilities for each class after combining prior knowledge and observed data. Hence, from (8), the Bayesian MMSE error estimator for an arbitrary classifier *ψ*_*n*_ is

(16) $$\begin{array}{l} \hat{\varepsilon }=\overset{b}{\underset{j=1}{\sum }}E_{\pi^{*}}[c]\frac{U_{j}+\alpha_{j}^{0}}{n_{0}+\sum_{i=1}^{b}\alpha_{i}^{0}}I_{\psi_{n}(j)=1}\\ \;+(1-E_{\pi^{*}}[c])\frac{V_{j}+\alpha_{j}^{1}}{n_{1}+\sum_{i=1}^{b}\alpha_{i}^{1}}I_{\psi_{n}(j)=0}, \end{array}$$

where **I**_*E*_ is an indicator function equal to one if *E* is true and zero otherwise. Exactly the same expression was derived using a brute-force approach in [27]. The optimal Bayesian classifier may now be found directly using (11):

(17) $$\psi_{\text{OBC}}(j)\;=\;\left\{\begin{array}{ll} 1\;\; & \text{if}\;E_{\pi^{*}}[\;c]\frac{U_{j}+\alpha_{j}^{0}}{n_{0}+\sum_{i=1}^{b}\alpha_{i}^{0}}\;<\;(1\;-\;E_{\pi^{*}}[\;c]\;)\frac{V_{j}+\alpha_{j}^{1}}{n_{1}+\sum_{i=1}^{b}\alpha_{i}^{1}},\\ 0\;\; & \text{otherwise}. \end{array}\right)$$

The optimal Bayesian classifier minimizes the Bayesian error estimator by minimizing each term in the sum (16). This is achieved by assigning *ψ*_OBC_(*j*) the class with the smaller constant scaling the indicator function. The expected error of the optimal classifier is

(18) $$\begin{array}{l} {\hat{\varepsilon }}_{\text{OBC}}=\overset{b}{\underset{j=1}{\sum }}\min\left\{E_{\pi^{*}}[c]\frac{U_{j}+\alpha_{j}^{0}}{n_{0}+\sum_{i=1}^{b}\alpha_{i}^{0}},\right)\\ \;\left((1-E_{\pi^{*}}[c])\frac{V_{j}+\alpha_{j}^{1}}{n_{1}+\sum_{i=1}^{b}\alpha_{i}^{1}}\right\}. \end{array}$$

In the special case where we have uniform *c* and uniform priors for the bin probabilities ($\alpha_{i}^{y}=1$ for all *i* and *y*), the Bayesian MMSE error estimate is

(19) $$\hat{\varepsilon }=\sum_{j=1}^{b}\frac{n_{0}+1}{n+2}\frac{U_{j}+1}{n_{0}+b}I_{\psi_{n}(j)=1}+\frac{n_{1}+1}{n+2}\frac{V_{j}+1}{n_{1}+b}I_{\psi_{n}(j)=0},$$

the optimal Bayesian classifier is

(20) $$\psi_{\text{OBC}}(j)=\left\{\begin{array}{ll} 1 & \text{if}\;\frac{n_{0}+1}{n_{0}+b}\left(U_{j}+1\right)<\frac{n_{1}+1}{n_{1}+b}\left(V_{j}+1\right),\\ 0 & \text{otherwise}, \end{array}\right)$$

and the expected error of the optimal classifier is

(21) $${\hat{\varepsilon }}_{\text{OBC}}=\overset{b}{\underset{j=1}{\sum }}\min\left\{\frac{n_{0}+1}{n+2}\frac{U_{j}+1}{n_{0}+b},\frac{n_{1}+1}{n+2}\frac{V_{j}+1}{n_{1}+b}\right\}.$$

Hence, under uniform priors, when the total number of samples observed in each class is the same (*n*_0_=*n*_1_), the optimal Bayesian classifier is equivalent to the classical discrete histogram rule, which assigns a class to each bin by a majority vote: *ψ*_DHR_(*j*)=1 if *U*_*j*_<*V*_*j*_ and *ψ*_DHR_(*j*)=0 if *U*_*j*_≥*V*_*j*_; otherwise, the discrete histogram rule is not necessarily optimal within an arbitrary Bayesian framework.

We take a moment to compare optimal Bayesian classification over an uncertainty class of distributions with Bayes classification for a fixed feature-label distribution. With fixed class-0 probability *c* and bin probabilities *p*_*i*_ and *q*_*i*_, the true error of an arbitrary classifier, *ψ*, is given by

(22) $$\varepsilon =\overset{b}{\underset{j=1}{\sum }}{\text{cp}}_{j}I_{\psi (j)=1}+(1-c)q_{j}I_{\psi (j)=0}.$$

Note a similarity to (16) and (19). The Bayes classifier is given by *ψ*_Bayes_(*j*)=1 if *cp*_*j*_<(1−*c*)*q*_*j*_ and zero otherwise, corresponding to (17) and (20). Finally, the Bayes error is given by

(23) $$\varepsilon_{\text{Bayes}}=\overset{b}{\underset{j=1}{\sum }}\min\{{\text{cp}}_{j},(1-c)q_{j}\},$$

corresponding to (18) and (21). Throughout, *c* corresponds to $E_{\pi^{*}}[c]$, *p*_*j*_ corresponds to the effective bin probability $f(j|0)=(U_{j}+\alpha_{j}^{0})/(n_{0}+\sum_{i=1}^{b}\alpha_{i}^{0})$ and similarly *q*_*j*_ corresponds to the effective bin probability *f*(*j*|1). In this case, the effective density is a member of our uncertainty class (which contains all possible discrete feature-label distributions), so that the optimal thing to do is simply plug the effective parameters in the fixed-distribution problem.

**Figure 4:**
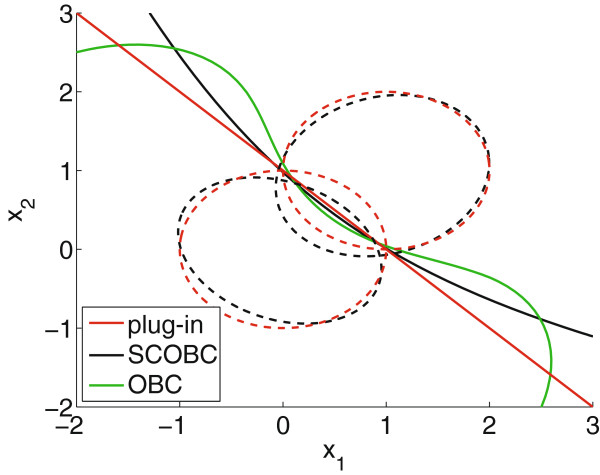
**Classifiers for an independent arbitrary covariance Gaussian model.** Classifiers for an independent arbitrary covariance Gaussian model with *D*=2 features and proper posteriors. Whereas the optimal Bayesian classifier (in green) is polynomial with expected true error 0.2007, the state-constrained optimal Bayesian classifier (in black) is quadratic with expected true error 0.2061 and the plug-in classifier (in red) is linear with expected true error 0.2078. These expected true errors are averaged over the posterior on the uncertainty class of states.

That being said, the effective density is not always a member of our uncertainty class. Consider an example with *D*=2 features, an uncertainty class of Gaussian class-conditional distributions with independent arbitrary covariances, and a proper posterior with fixed class-0 probability *c*=0.5 (hyperparameters are provided in [32]). We consider three classifiers. First is a plug-in classifier, which is the Bayes classifier corresponding to the posterior expected parameters, *c*=0.5, *μ*_0_= [0,0,…,0], *μ*_1_= [1,1,…,1], and *Σ*_0_=*Σ*_1_=*I*_*D*_. Since the expected covariances are homoscedastic, this classifier is linear. The second is a state-constrained optimal Bayesian classifier, *ψ*_SCOBC_, in which we search for a state with corresponding Bayes classifier having smallest expected error over the uncertainty class [34]. Since the Bayes classifier for any particular state in the uncertainty class is quadratic, this classifier is quadratic. Finally, we have the optimal Bayesian classifier, which has been solved analytically in [29], although details are omitted here. In this case, the effective densities are not Gaussian but multivariate student’s *t* distributions, resulting in an optimal Bayesian classifier having a polynomial decision boundary that is higher than quadratic order. Figure 4 shows *ψ*_plug−in_ (red), *ψ*_SCOBC_ (black) and *ψ*_OBC_ (green). Level curves for the class-conditional distributions corresponding to the expected parameters used in *ψ*_plug−in_ are shown in red dashed lines, and level curves for the distributions in the state corresponding to *ψ*_SCOBC_ are shown in black dashed lines. These were found by setting the Mahalanobis distance to 1. Each classifier is quite distinct, and in particular, the optimal Bayesian classifier is non-quadratic even though all class-conditional distributions in the uncertainty class are Gaussian.

**Figure 5:**
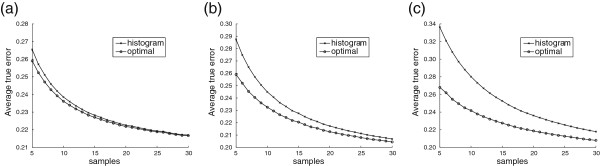
**Average true errors for discrete classification.** Average true errors on discrete distributions from known priors with uniform *c* and bin probabilities versus sample size. **(a)***b*=2; **(b)***b*=4; **(c)***b*=8.

To demonstrate the performance advantage of optimal Bayesian classification via a simulated experiment, we return to the discrete classification problem. Let *c* and the bin probabilities be generated randomly according to uniform prior distributions. For each fixed feature-label distribution, a binomial (*n*,*c*) experiment is used to determine the number of sample points in class 0 and the bin for each point is drawn according to the bin probabilities corresponding to its class, thus generating a non-stratified random sample of size *n*. Both the histogram rule and the new optimal Bayesian classifier from (20), assuming correct priors, are trained from the sample. The true error for each classifier is also calculated exactly via (22). This is repeated 100,000 times to obtain the average true error for each classification rule, presented in Figure 5 for *b*=2, 4 and 8 bins. Observe that the average performance of optimal Bayesian classification is indeed superior to that of the discrete histogram rule, especially for larger bin sizes. However, note that optimal Bayesian classifiers are not guaranteed to be optimal for a specific distribution (the optimal classifier is the Bayes classifier), but only optimal when averaged over all distributions in the assumed Bayesian framework.

## Conclusions

Scientific knowledge is possible for small-sample classification.

Given the importance of classification throughout science and the crucial epistemological role played by error estimation, it is remarkable that only one paper providing analytic results for moments of common error estimators was published between 1977 and 2005, and that up until 2005, there were no papers providing representation of the joint distribution or of the second-order mixed moments. Today, we are paying the price for this dearth of activity as we are now presented with very large feature sets and small samples across different disciplines, in particular, in high-throughput biology, where the advance of medical science is being hamstrung by a lack of basic knowledge regarding pattern recognition. Moreover, in spite of this obvious crippling lack of knowledge, there is only a minuscule effort to rectify the situation, whereas billions of dollars are wasted on gathering an untold quantity of data that is useless absent the requisite statistical knowledge to make it useful.

No doubt this unfortunate situation would make for a good sociological study. But that is not our field of expertise. Nonetheless, we will put forth a comment made by Thomas Kailath in 1974, about the time that fundamental research in error estimation for small-sample classification came to a halt. He writes, “It was the peculiar atmosphere of the sixties, with its catchwords of ‘building research competence,’ ‘training more scientists,’ etc., that supported the uncritical growth of a literature in which quantity and formal novelty were often prized over significance and attention to scholarship. There was little concern for fitting new results into the body of old ones; it was important to have ‘new’ results!” [35]. Although Kailath’s observation was aimed at signal processing, the “peculiar atmosphere” of which he speaks is not limited to any particular discipline; rather, he had perceived an “uncritical growth of a literature” lacking “attention to scholarship.” One can only wonder what Prof. Kailath’s thoughts are today when he surveys a research landscape that produces orders of magnitude more papers but produces less knowledge than that produced by the relative handful of scientists, statisticians, and engineers a half century ago. For those who would question this latter observation in pattern recognition, we suggest a study of the early papers by such pioneers as Theodore Anderson, Albert Bowker, and Rosedith Sitgreaves.

## Competing interests

Both authors declare that they have no competing interests.

## References

[B1] Dougherty ER Braga-Neto U Epistemology of computational biology: mathematical models and experimental prediction as the basis of their validity J. Biol. Syst 2006 14 65 90 10.1142/S0218339006001726

[B2] Reichenbach H The Rise of Scientific Philosophy 1971 Berkeley: University of California Press

[B3] Dougherty ER Bittner ML Epistemology of the Cell: A Systems Perspective on Biological Knowledge. IEEE Press Series on Biomedical Engineering 2011 New York: John Wiley

[B4] Feynman R QED: The Strange Theory of Light and Matter 1985 Princeton: Princeton University Press

[B5] Frank P Modern Science and Its Philosophy 1961 New York: Collier Books

[B6] Dougherty ER On the epistemological crisis in genomics Curr. Genomics 2008 9 2 69 79 10.2174/138920208784139546 19440447 PMC2674806

[B7] Brun M Sima C Hua J Lowey J Carroll B Suh E Dougherty ER Model-based evaluation of clustering validation measures Pattern Recognit 2007 40 3 807 824 10.1016/j.patcog.2006.06.026

[B8] Devroye L Györfi L Lugosi G A Probabilistic Theory of Pattern Recognition. Stochastic Modelling and Applied Probability 1996 New York: Springer

[B9] Dougherty ER Biomarker development: prudence, risk, and reproducibility BioEssays 2012 34 4 277 279 10.1002/bies.201200003 22337590

[B10] Hanczar B Hua J Dougherty ER Decorrelation of the true and estimated classifier errors in high-dimensional settings EURASIP J. Bioinformatics Syst. Biol 2007 2007 12 Article ID 38473 10.1155/2007/38473 PMC3171336 18288255

[B11] Braga-Neto U ER Dougherty, Exact performance of error estimators for discrete classifiers Pattern Recognit 2005 38 11 1799 1814 10.1016/j.patcog.2005.02.013

[B12] Hills M Allocation rules and their error rates J. R. Stat. Soc. Ser. B (Stat. Methodology) 1966 28 1 31

[B13] Foley D Considerations of sample and feature size IEEE Trans. Inf. Theory 1972 18 5 618 626 10.1109/TIT.1972.1054863

[B14] Sorum MJ Estimating the conditional probability of misclassification Technometrics 1971 13 333 343 10.1080/00401706.1971.10488788

[B15] McLachlan GJ An asymptotic expansion of the expectation of the estimated error rate in discriminant analysis Aust 1973 15 3 210 214 10.1111/j.1467-842X.1973.tb00201.x

[B16] Moran M On the expectation of errors of allocation associated with a linear discriminant function Biometrika 1975 62 141 148 10.1093/biomet/62.1.141

[B17] Goldstein M Wolf E On the problem of bias in multinomial classification Biometrics 1977, 1975 33 325 331

[B18] Davison A Hall P On the bias and variability of bootstrap and cross-validation estimates of error rates in discrimination problems Biometrica 1992 79 274 284

[B19] Xu Q Hua J Braga-Neto UM Xiong Z Suh E Dougherty ER Confidence intervals for the true classification error conditioned on the estimated error Technol. Cancer Res. Treat 2006 5 579 590 17121434 10.1177/153303460600500605

[B20] Zollanvari A Braga-Neto UM Dougherty ER On the sampling distribution of resubstitution and leave-one-out error estimators for linear classifiers Pattern Recognit 2009 42 11 2705 2723 10.1016/j.patcog.2009.05.003

[B21] Zollanvari A Braga-Neto UM Dougherty ER On the joint sampling distribution between the actual classification error and the resubstitution and leave-one-out error estimators for linear classifiers IEEE Trans Inf. Theory 2010 56 2 784 804

[B22] Zollanvari A Braga-Neto UM Dougherty ER Exact representation of the second-order moments for resubstitution and leave-one-out error estimation for linear discriminant analysis in the univariate Heteroskedastic Gaussian Model Pattern Recognit 2012 45 2 908 917 10.1016/j.patcog.2011.08.006

[B23] Zollanvari A Braga-Neto UM Dougherty ER Analytic study of performance of error estimators for linear discriminant analysis IEEE Trans. Signal Process 2011 59 9 4238 4255

[B24] Wyman F Young D Turner D A comparison of asymptotic error rate expansions for the sample linear discriminant function Pattern Recognit 1990 23 775 783 10.1016/0031-3203(90)90100-Y

[B25] Pikelis V Comparison of methods of computing the expected classification errors Automatic Remote Control 1976 5 59 63

[B26] Dougherty ER Zollanvari A Braga-Neto UM The illusion of distribution-free small-sample classification in genomics Curr. Genomics 2011 12 5 333 341 10.2174/138920211796429763 22294876 PMC3145263

[B27] Dalton LA Dougherty ER Bayesian minimum mean-square error estimation for classification error–part I: definition and the Bayesian MMSE error estimator for discrete classification IEEE Trans. Signal Process 2011 59 115 129

[B28] Dalton LA Dougherty ER Exact sample conditioned MSE performance of the Bayesian MMSE estimator for classification error–part I: representation IEEE Trans. Signal Process 2012 60 5 2575 2587

[B29] Dalton LA Dougherty ER Optimal classifiers with minimum expected error within a, Bayesian framework–part I: discrete and Gaussian models Pattern Recognit 2013 46 5 1301 1314 10.1016/j.patcog.2012.10.018

[B30] Dalton LA Dougherty ER Application of the Bayesian MMSE estimator for classification error to gene expression microarray data Bioinformatics 2011 27 13 1822 1831 10.1093/bioinformatics/btr272 21551140

[B31] Dalton LA Dougherty ER Exact sample conditioned MSE performance of the Bayesian MMSE estimator for classification error–part II: consistency and performance analysis IEEE Trans. Signal Process 2012 60 5 2588 2603

[B32] Dalton LA Dougherty ER Optimal classifiers with minimum expected error within a Bayesian framework–part II: properties and performance analysis Pattern Recognit 2013 46 5 1288 1300 10.1016/j.patcog.2012.10.019

[B33] Dalton LA Dougherty ER Bayesian minimum mean-square error estimation for classification error–part II: the Bayesian MMSE error estimator for linear classification of Gaussian distributions IEEE Trans. Signal Process 2011 59 130 144

[B34] Dougherty ER Hua J Xiong Z Chen Y Optimal robust classifiers Pattern Recognit 2005 38 10 1520 1532 10.1016/j.patcog.2005.01.019

[B35] Kailath T A view of three decades of linear filtering theory IEEE Transact. Inf. Theory 1974 20 2 146 181 10.1109/TIT.1974.1055174

